# Screening and Validation of Reference Genes for RT-qPCR Under Different Honey Bee Viral Infections and dsRNA Treatment

**DOI:** 10.3389/fmicb.2020.01715

**Published:** 2020-07-30

**Authors:** Yanchun Deng, Hongxia Zhao, Sa Yang, Li Zhang, Lina Zhang, Chunsheng Hou

**Affiliations:** ^1^Institute of Apicultural Research, Chinese Academy of Agricultural Sciences, Beijing, China; ^2^Key laboratory of Pollinating Insect Biology, Ministry of Agriculture and Rural Affairs, Beijing, China; ^3^Graduate School of Chinese Academy of Agricultural Sciences, Beijing, China; ^4^Guangdong Key Laboratory of Animal Conservation and Resource Utilization, Guangdong Public Laboratory of Wild Animal Conservation and Utilization, Guangdong Institute of Applied Biological Resources, Guangdong Academy of Science, Guangzhou, China

**Keywords:** honey bee, RT-qPCR, reference genes, bee virus, dsRNA treatment

## Abstract

Honey bee viruses are one of the most important pathogens that have contributed to the decrease in honey bee colony health. To analyze the infection dynamics of honey bee viruses, quantification of viral gene expression by RT-qPCR is necessary. However, suitable reference genes have not been reported from viral and RNAi studies of honey bee. Here, we evaluated the expression of 11 common reference genes (*ache*2, *rps*18, β*-actin*, *tbp*, *tif*, *rpl*32, *gadph*, *ubc*, α*-tubulin*, *rpl14*, and *rpsa*) from *Apis mellifera* (Am) and *Apis cerana* (Ac) under Israeli acute paralysis virus (IAPV), chronic bee paralysis virus (CBPV), and Chinese sacbrood virus (CSBV) infection as well as dsRNA-PGRP-SA treatment, and we confirmed their validation by evaluating the levels of the *defensin 1* and prophenoloxidase (*ppo*) genes during viral infection. Our results showed that the expression of selected genes varied under different viral infections. *ache*2, *rps*18, β*-actin*, *tbp*, and *tif* can be used to normalize expression levels in *Apis mellifera* under IAPV infection, while the combination of *actin* and *tif* is suitable for CBPV-infected experiments. The combination of *rpl*14, *tif*, *rpsa*, *ubc*, and *ache*2 as well as more reference genes is suitable for CSBV treatment in *Apis cerana*. *Rpl*14, *tif*, *rps*18, *ubc*, and α*-tubulin* were the most stable reference genes under dsRNA treatment in *Apis mellifera*. Furthermore, the geNorm and NormFinder algorithms showed that *tif* was the best suitable reference gene for these four treatments. This study screened and validated suitable reference genes for the quantification of viral levels in honey bee, as well as for RNAi experiments.

## Introduction

Honey bees provide a significant pollination service for many agricultural crops and wild plants. However, a decline in bee populations has been recently observed in the United States and parts of European countries (Brutscher et al., 2015). Although multiple biotic and abiotic factors contribute to colony decline, several epidemiological and temporal monitoring studies have indicated that pathogens play an important role in colony loss (Brutscher et al., 2015). Among these pathogens, RNA viruses are the major impacting factors, including two *Apis mellifera* viruses, Israeli acute bee paralysis virus (IAPV) (Maori et al., 2007) and chronic bee paralysis virus (CBPV) (Genersch and Aubert, 2010), as well as one *Apis cerana* virus, namely, Chinese sacbrood virus (CSBV) (Li et al., 2018).

Israeli acute paralysis virus, a positive-sense RNA virus in the family *Dicistroviridae*, has a widespread impact on honey bee health and has been linked with colony losses (Chen et al., 2014; Galbraith et al., 2015). RNA-seq data indicated that IAPV attacks several core genes in insect immune pathways (Toll, JAK-STAT and RNA interference pathways) (Galbraith et al., 2015). Although CBPV has not been classified, its genome, which is made up of two segments of single-stranded RNA in a non-enveloped anisometric capsid, shows homology to the Nodaviridae and Tombusviridae families (Coulon et al., 2017). Exposure of honey bees to CBPV alone promoted downregulation of immune-related genes, such as *dorsal-1a* and *ppo* (Coulon et al., 2017). CSBV, a geographic strain of SBV isolated from *A. cerana* in China, can cause fatal infections to *A. cerana* larvae by decreasing antimicrobial peptide levels (Shan et al., 2017). The quantification of viral and immune gene expression levels is one of the most important aspects when studying host and virus interactions (Marcial-Quino et al., 2016). RNA interference (RNAi) technology is frequently used as a research tool to study the function, transcription and regulation of gene including immune genes (Vyas et al., 2017; Zhu et al., 2019). Introducing exogenous double stranded RNA (dsRNA) into the insect cells activates RNAi pathways that normally function to induce antiviral responses (Dietrich et al., 2017). To analyze the expression differences in immune genes during viral infection and RNAi process, RT-qPCR is used to assess gene expression by normalization with reference genes. Most gene expression studies focused on honey bee select *actin* as an internal control (Chen et al., 2014), though RPS5 (Wheeler et al., 2006) and EIF S8 (Hunt et al., 2007) are also occasionally used. However, the identification of reference genes was not made in advance when these studies assessed viral dynamics and the changes in corresponding target genes.

As summarized by Shakeel et al. (2018), the identification and validation of reference genes for qRT-PCR have been performed in various insect species. For honey bee, the stability of reference genes has been investigated in different developmental stages and tissues (Lourenco et al., 2008; Reim et al., 2013; Moon et al., 2018a), and even after bacterial challenge (Scharlaken et al., 2008). However, suitable candidate reference genes have not been validated during honey bee viral infection. Moreover, most of these genes displayed variable expression levels under different experimental conditions (Thellin et al., 2009).

Thus, we evaluated the expression of 11 reference genes under infection with three different viruses and under experimental RNAi conditions to identify the most suitable reference genes for different conditions. Here, 11 reference genes, including *A. mellifera ribosomal protein L32* (*Amrpl*32), *A. mellifera 40S ribosomal protein S18* (*Amrps*18), *A. mellifera TATA-box-binding protein* (*Amtbp*), *A. mellifera*α*-tubulin alpha-1C chain* (*Am*α*-tubulin*), *A. mellifera glyceraldehyde-3-phosphate dehydrogenase 2* (*Amgadph*), *A. mellifera acetylcholinesterase 2* (*Amache*2), *A. mellifera translation initiation factor eIF-2B subunit delta* (*Amtif*), *A. mellifera ubiquitin-conjugating enzyme E2-22 kDa* (*Amubc*), *A. mellifera actin related protein 1* (*Am*β*-actin*), *Apis cerana 60S ribosomal protein L14* (*Acrpl*14), *Apis cerana 40S ribosomal protein SA* (*Acrpsa*), *Actbp*, *Ac*α*-tubulin*, *Acgadph*, *Acache2*, *Actif*, *Acubc*, and *Ac*β*-actin*, were analyzed in *A. mellifera* under IAPV or CBPV infection as well as RNAi experimental conditions. The corresponding genes from *A. cerana* were also used to determine the most stable reference genes for RT-qPCR under CSBV infection. Five common algorithms, including NormFinder, BestKeeper, Delta-Ct, geNorm, and RefFinder, were used to evaluate reference gene expression. We also tested the reliability of the reference genes by investigating the expression levels of two immune genes, *defensin 1* and prophenoloxidase (*ppo*), under different viral infections. We hope that this report will provide a basis for future studies on gene expression in honey bee under viral infection.

## Materials and Methods

### Collection of Virus-Infected Honey Bees

Israeli acute paralysis virus-infected *A. mellifera* adults with obvious paralysis symptom (Maori et al., 2007) were collected from three different apiaries in Beijing, Miyun, China, while CBPV-infected *A. mellifera* adults with paralysis symptom (Olivier et al., 2008) were collected from three different apiaries in Guangdong Province in China. They were tested by RT-PCR with special primers (Diao et al., 2018). Healthy *A. cerana* larvae were collected from three different apiaries in Guangdong Province in China. CSBV-infected larvae were taken from naturally infected colonies with obvious cystic phenotypes and symptoms. The samples were immediately transported to the laboratory on dry ice to avoid the degradation of the active substances therein.

We collected seemingly healthy bee samples from the experimental apiary at the Institute of Apicultural Research (IAR), Chinese Academy of Agricultural Sciences, Beijing, China. Newly emerged bees (*A. mellifera*) were obtained from brood frames taken from the experimental honey bee hives and maintained in an incubator at 30°C and 60% relative humidity (RH) for approximately 12 h with access to a 50% sucrose solution. Honey bee samples were divided into five groups with three repetitions per group with 30 bees. The first, second, and third groups consisted of bees treated with IAPV, CBPV, and CSBV, respectively; the fourth group consisted of bees treated with dsRNA-pgrpsa; and the fifth group was used to test the reliability of the reference genes. Each group contained a blank control, except for the dsRNA experiment, which had dsRNA-GFP as a control.

To gain reliable samples, PCR detection was performed. Samples in IAPV group were collected every day (24, 48, 72, and 96 h) and followed by PCR using IAPV primers (Supplementary Table S1). Similarly, samples in CBPV group were collected every day (0, 24, 48, 72, and 96 h) and followed by PCR using CBPV primers (Supplementary Table S1). The naturally CSBV-infected larvae and the healthy larvae of *A. cerana* were collected from 2th to 6th-instar and followed by PCR using CSBV primers (Supplementary Table S1). Samples in dsRNA treatment group were collected every day (24, 48, 72, and 96 h) and followed by PCR using PGRP-SA primers (Supplementary Table S1).

### Purification and Inoculation of IAPV and CBPV

To purify the viruses, the IAPV (IAPV-BJ, KX421583.1) and CBPV (CBPV-HB, MF175174.1, and MF175173.1) field strains originating from paralyzed bees showing clinical symptoms of viral infection were collected in May 2017 in China. IAPV and CBPV were screened for predominance in the samples, as well as the absence of other common viruses. IAPV was purified as described by Maori et al. (2007). CBPV was purified from the heads of bees by ultracentrifugation in a 10 to 40% (w/v) sucrose gradient as previously described by Olivier et al. (2008). To avoid interference by other viruses, PCR was run to identify whether there was infection by four other common honey bee viruses before virus purification (Supplementary Table S1), such as black queen cell virus (BQCV), acute bee paralysis virus (ABPV), deformed wing virus (DWV) and aphid lethal paralysis virus (ALPV) following the method of Diao et al. (2018) and Yang et al. (2019). The purified virus particles were dissolved in sucrose at −80°C and aliquoted until use.

Purified virus particles (5 μl, 1 μg/μl) with about 4.0 × 10^4^ genome equivalent copies was injected into the abdominal intersegment space of 30 individual newly emerged bees taken from a healthy colony. For the IAPV and CBPV infection groups, 5 μg of IAPV and CBPV, respectively, was injected into newly emerged honey bees (*A. mellifera*). The naturally infected CSBV and normal control *A. cerana* larvae were collected from the 2nd to 6th instars. For the dsRNA group, dsRNA-PGRP-SA and dsRNA-GFP (5 μg) were injected into newly emerged honey bees (*A. mellifera*) at 0 and 48 h, respectively. The honey bee samples were detected for six other viruses before they were used for the infection experiments. The infected bees were maintained in an incubator at 30°C, and their mortality was calculated each day. Bees in control group were injected with PBS buffer.

### RNA Extraction and RT-qPCR

Total RNA was extracted with the TRIol Kit (Ambion, Life Technologies, United States) following the manufacturer’s instructions. A NanoDrop 2000 (Thermo Scientific, United States) was used to check the quality of each RNA sample. RNA samples with A260/280 ratios ranging from 1.8 to 2.2 were used for cDNA synthesis with the PrimeScript RT Reagent Kit with gDNA Eraser (Takara, Dalian, China) according to the manufacturer’s instructions. The cDNAs of all samples were stored at −20°C until use.

The specific primers for the reference genes were designed using Primer-BLAST (Table 1). The RT-qPCR reaction system consisted of 12.5 μL 2 × SYBR Premix Ex Taq^TM^ II (Takara, Dalian, China), 0.5 μL each of the forward and reverse primers (10 mM), 2 μL cDNA template, and 9.5 μL double-distilled H_2_O for a total volume of 25 μL. The RT-qPCR conditions followed the manufacturer’s instructions. All reactions were performed using SYBR Green Premix and amplified under the following cycling conditions: an initial cycle at 95°C; 40 cycles of 95°C for denaturation, 25 s at 55°C for annealing and 20 s at 72°C for extension; and the generation of a melting curve consisting of a single peak to rule out non-specific products and primer dimers afterward. The RT-qPCR analysis was performed with three biological replicates for each sample and three technical replicates for each biological replicate and was measured as the mean Ct value. The results were analyzed using the 9600 plus Software.

**TABLE 1:** Primers used for RT-qPCR of the candidate reference genes.

**Gene name**	**Gene symbol**	**Primer sequence (5′–3′)**	**Amplicon size (bp)**	**GenBank accession no.**
*Apis mellifera ribosomal protein L32*	*Amrpl32*	F:CGTCACCAGAGTGATCGTTACA	244	NM_001011587.1
		R:CCCCATGAGCAATTTCAGCAC		
*Apis mellifera 40S ribosomal protein S18*	*Amrps18*	F:GGTGTTGGTCGTCGTTATGC	269	XM_625101.6
		R:CGCAAACCTCTATGAGCACG		
*Apis mellifera TATA-box-binding protein*	*Amtbp*	F:GCAACCTCAAACACCGCAAA	121	XM_623085.6
		R:TGCAGAAGCCGGTGTCATAG		
*Apis mellifera tubulin alpha-1C chain*	*Am*α-*tubulin*	F:TGAGTCAGACAGTAGTCGGAT	126	XM_006558697.3
		R:TACCCATTTGGACACCAGCC		
*Apis mellifera glyceraldehyde-3-phosphate dehydrogenase 2*	*Amgadph*	F:TACCGCTTTCTGCCCTTCAA	142	XM_393605.7
		R:GCACCGAACTCAATGGAAGC		
*Apis mellifera acetylcholinesterase 2*	*Amache2*	F:GACGCGAAGACCATATCCGT	140	NM_001040230.1
		R:TCTGTGTCCTTGAAGTCCGC		
*Apis mellifera translation initiation factor eIF-2B subunit delta*	*Amtif*	F:TGAAACAAGAGGAACATGTCTAA	121	XM_006559190.3
		R:CGTTCTGCTTTTATTTCCTCCC		
*Apis mellifera ubiquitin-conjugating enzyme E2-22 kDa*	*Amubc*	F:TCAAAAGGGTGAACACGAGC	208	XM_016917677.1
		R:AGTTCGCTGAATGCGTCAAC		
*Apis mellifera actin related protein 1*	*Amactin*	F:TTGTATGCCAACACTGTCCTT	120	NM_001185146.1
		R:TGGCGCGATGATCTTAATTT		
*Apis cerana 60S ribosomal protein L14*	*Acrpl14*	F:TTGCTTCCGGTAAACCATGC	261	XM_017051728.2
		R:ATGTTTGCAGCTTCCCATGC		
*Apis cerana 40S ribosomal protein SA*	*Acrpsa*	F:TTGGAAGTCTCCTAGTGGCA	314	XM_017053220.2
		R:GCTCTTTGTCCAGTTTGTCGG		
*Apis cerana TATA-box-binding protein*	*Actbp*	F:AAGTCCGATGACCCCAGCTA	302	XM_028665297.1
		R:TAGCTGCAAAACCTAACTTCTGAAT		
*Apis cerana tubulin alpha-1 chain*	*Actubulin*	F:GTTCGACTGTGCGTTGTGTG	211	XM_017057619.2
		R:TGACCATCAGGTTGGATGCC		
*Apis cerana glyceraldehyde-3-phosphate dehydrogenase 2*	*Acgadph*	F:CTGCACAGACCCGAGTGAAT	120	XM_017062468.2
		R:GCAACAACCTGAGCACCAAA		
*Apis cerana acetylcholinesterase*	*Acache*2	F:CTGGTCTTGGTAAAGGGCCA	303	XM_017066417.2
		R:CCTGGACGAAGCCCTAACAC		
*Apis cerana translation initiation factor eIF-2B subunit delta*	*Actif*	F:TGGAACTGCTCAAGTTGCCT	241	XM_028664590.1
		R:ATCGGCTGGTGTAACATCGT		
*Apis cerana ubiquitin-conjugating enzyme E2-22 kDa*	*Acubc*	F:GCTGCTGCCATGACTTTACG	280	XM_017048857.2
		R:TAGCGCGTTCCAAATCCCAA		
*Apis cerana actin*	*Acacetin*	F:CGAGCACGGTATCATCACCA	329	JX899419.1
		R:CCAAGTCCAGACGGAGGATG		
*Apis mellifera defensin 1*	*AmDef1*	F:AAGAACGTGCCGACAGACAT	121	NM_001011616.2
		R:TCGCAATGACCTCCAGCTTT		
*Apis mellifera phenoloxidase subunit A3*	*Amppo*	F:AGATGGCATGCATTTGTTGA	332	NM_001011627.1
		R:CCACGCTCGTCTTCTTTAGG		
*Apis cerana defensin 1*	*AcDef1*	F: AATTCGAGCCACTTGAGCATCC	148	XM_017050425.2
		R: CAATGACCTCCAGCTTTACCCAA		

### PCR Detection for Viral and Immune Genes

The primer sequences, orientation and references are provided in Supplementary Table S1. The cDNAs of all samples were gained in the above, followed by the PCR cycles. The PCR reaction consisted of a total 20 μL volume containing 10 μL 2 × GoTaq reaction buffer (Promega, United States), 0.5 μL 10 μM of the sense and antisense primers, 1 μL of cDNA, and 8 μL nuclease-free water. The cycling conditions were as follows: 1 min at 95°C; 33 cycles of 30 s at 94°C, 30 s at 55 and 72°C for 1 min; a final extension of 10 min at 72°C; and cooling to 4°C. The PCR amplification products were separated in a 2% agarose gel stained with GV II (BIOMEC, China) and photographed with a FR-200A luminescent and fluorescent biological image analysis system (Furi, China). The product size was determined using a 100-bp molecular size ladder.

### dsRNA Synthesis

The specific primers for PGRP-SA and GFP tagged with T7 promoter sequence were designed using Primer-BLAST to amplify the templates for synthesis of dsRNA (Supplementary Table S2). Amplification was performed under normal PCR conditions as follows: 30 s at 95°C; followed by 35 cycles of 8 s at 95°C, 30 s at 56°C, and 30 s at 72°C; and a final extension incubation at 72°C for 5 min. The PCR templates were purified using a Qiagen purification kit (Qiagen, Germantown, MD, United States). dsRNA-PGRP-SA and dsRNA-GFP were produced using a T7 RNAi Transcription Kit (Vazyme, China) according to the manufacturer’s instructions. The reaction product was subjected to DNase and RNAse digestion, and then incubated at 37°C for 8 h. Subsequently, the samples were dried at 37°C for 10 min and resuspended in 20–40 μL of nuclease-free H_2_O. The quality of the dsRNA was checked by electrophoresis and quantified with a spectrophotometer (NanoDrop Technologies, Wilmington, DE, United States). Then, 5 μg each of PGRPSA dsRNA and *GFP* dsRNA was injected into newly emerged *A. mellifera* at 0 and 48 h, respectively.

### Stability of Expression of Selected Reference Genes

The stability of expression of the candidate reference genes was evaluated by five statistical algorithms, namely, the delta CT method (Silver et al., 2006), BestKeeper (Pfaffl et al., 2004), geNorm (Vandesompele et al., 2002), NormFinder (Andersen et al., 2004), and the online platform RefFinder (Xie et al., 2012). geNorm is based on the principle that the expression ratio of two reference genes should be constant in all samples, regardless of the experimental conditions or sampling time (Hellemans et al., 2007). The gene expression stability (M) is defined as the average pairwise variation compared with all other tested candidate genes by geNorm, and the reference genes with the lowest *M*-value remained (Vandesompele et al., 2002). Pairwise variation (Vn/Vn + 1) analysis between the normalization factors (NFn and NFn + 1) was performed by geNorm to determine the optimal number of reference genes (Vandesompele et al., 2002). NFn was also calculated by the stepwise inclusion of a less stable gene until the (*n* + 1)th gene had no significant contribution to the newly calculated NFn + 1 (Vandesompele et al., 2002). In particular, if the pairwise variation Vn/n + 1 between the two sequential normalization factors NFn and NFn + 1 was lower than the cut-off value of 0.15, it was considered that NFn + 1 is not required (Peng et al., 2018). NormFinder was used to estimate changes and intra- and intergroup expression variation among the candidate reference genes (Andersen et al., 2004). The most stable gene is the one with the lowest stability value according to the intra- and intergroup variability of each gene (Andersen et al., 2004). BestKeeper determines the most stably expressed genes according to the standard deviation (SD) and coefficient of variation (CV) of all the Ct values for each gene (Pfaffl et al., 2004). When the SD values of genes are <1, they are considered stable (Marcial-Quino et al., 2016). Similarly, the delta-Ct method was used to identify suitable housekeeping genes based on the average SD value of each gene (Silver et al., 2006). To identify the optimal reference genes, RefFinder, a user-friendly web-based comprehensive tool that provides a comprehensive ranking evaluation based on the geometric mean and integrates with other computational algorithms (geNorm, NormFinder, BestKeeper, and the comparative Delta-Ct method), was used to reduce bias or avoid contradictory results caused by using individuals (Peng et al., 2018). Individual genes were assigned an appropriate weight and the geometric mean of their weights was calculated for the overall final ranking based on each program.

### Validation of the Selected Reference Genes

The three strategies for expression profile normalization using the reference genes in each treatment are as follows: (1) the optimal reference gene from all the samples, (2) the optimal reference gene from each treatment, and (3) the least stable reference gene from each treatment.

To validate the candidate reference genes, the relative expression levels of two immune genes (*defensin1* and *prophenoloxidase*) were used to evaluate the reference genes by RT-qPCR. The 2^–ΔΔ*CT*^ method was employed to validate and quantify the expression levels of *def1* and *ppo* after viral infection. One-way ANOVA was used to analyze the significance and the means were compared using Tukey’s multiple comparison test (*P* < 0.05) using the GraphPad Prism software.

## Results

### Identification of Primer Specificity and the Efficiency of the Candidate Reference Genes

**FIGURE 1:**
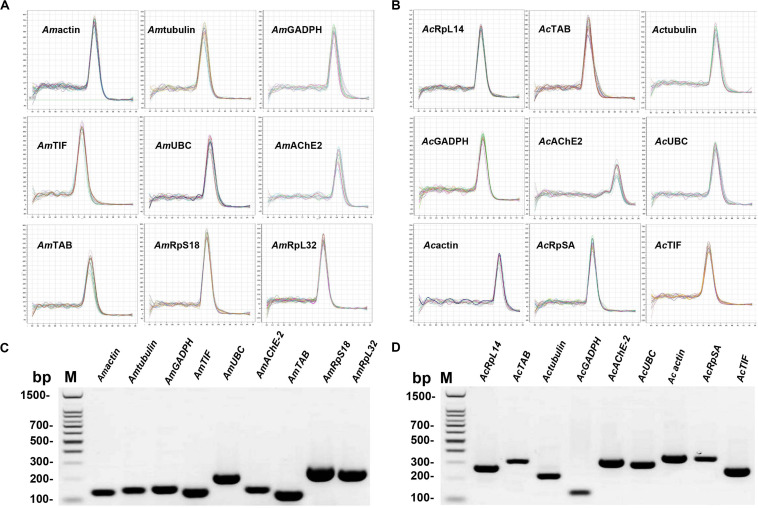
Confirmation of primer specificity and amplicon size. **(A)** Melting curve analysis of the *Apis mellifera* candidate reference genes. All RT-qPCR products had a single melting curve indicating the breakdown of only one PCR product. **(B)** Melting curve analysis of *Apis cerana* candidate reference genes. All RT-qPCR products had a single melting curve indicating only one PCR product. **(C)** Amplification results for *Apis mellifera* candidate genes from cDNA template. **(D)** Amplification results for *Apis cerana* candidate genes from cDNA template. M: DL1500 DNA marker.

The reference gene candidates in this study were selected based on the following two criteria: (a) genes routinely used in honey bees or insects for transcript normalization and (b) genes described as stably expressed under environmental stress. Eleven candidate reference genes were selected for *A. mellifera* and *A. cerana* under different treatments. The amplicon sizes of the target genes were between 100 and 320 bp with primer lengths of 17–25 bp and a 40–60% GC content. Primer specificity for each gene was validated by melting curve analysis during qRT-PCR, and a single peak was observed in each reaction (Figures 1A,B). Primer specificity for all genes was further tested using 2.0% agarose gels. A single PCR amplification band of the expected size was observed, suggesting that primer dimers and non-specific amplified products were not generated (Figures 1C,D). Additionally, the amplification efficiencies ranged from 92.2 to 107% based on the standard curves for the 18 genes. Moreover, correlation coefficients (*R*^2^) were greater than 0.99, which fit the RT-qPCR requirements (Supplementary Table S3).

### Characterization of Candidate Reference Genes

To analyze the expression profiles of the candidate reference genes, sample validation was performed by PCR (Supplementary Table S1). As shown in Supplementary Figure S1, the samples from the IAPV-treated group were positive but the control groups were not (Supplementary Figure S1A). Similarly, positive results were also observed for the CBPV and CSBV groups (Supplementary Figures S1B,C). The results for the dsRNA treatment groups showed that the expression of PGRP-SA had been markedly downregulated compared to the control group (Supplementary Figure S1D).

**FIGURE 2:**
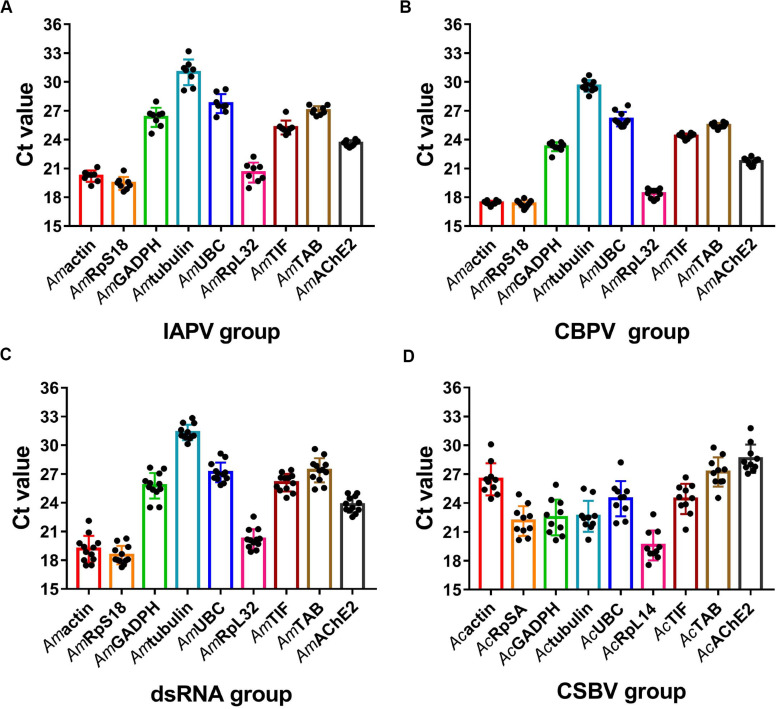
Expression profiles of candidate reference genes in different groups. Scatter plot analysis showing the raw Ct values of the candidate reference genes in **(A)** IAPV-infected honey bee. **(B)** CBPV-infected honey bee. **(C)** honey bee exposed to dsRNA PGRPSA treatment and **(D)** CSBV-infected *A. cerana* larvae.

A reliable reference gene should show constant expressions level under different experimental conditions (Galli et al., 2015). We next evaluated the expression levels of the candidate reference genes and found that the average Ct values of the reference genes in different samples ranged from 16 to 32 (Figure 2). In the *A. mellifera* groups, RPS18 was the most abundant with the lowest mean Ct value at 19.43 ± 0.67 in the IAPV-treated group (Figure 2A), 17.31 ± 0.37 in the CBPV-treated group (Figure 2B) and 18.52 ± 0.96 in the dsRNA-treated group (Figure 2C). In contrast, *Am*α*-tubulin* transcription showed the lowest level with the highest mean Ct value at 30.99 ± 1.33 in the IAPV-treated group, 29.58 ± 0.62 in the CBPV-treated and 31.33 ± 0.80 in the dsRNA-treated group (Figure 2). In the *A. cerana* group, *ache*2 exhibited the smallest SD value (28.6 ± 1.47) but had the lowest expression level (Figure 2D). In contrast, *rpl*14 exhibited the smallest Ct value (19.59 ± 1.56) but had a larger SD value. *Tif* was a moderately stable gene with Ct average values at 25.25 ± 0.73 in the IAPV-treated group, 24.38 ± 0.26 in the CBPV-treated group, 26.10 ± 0.9 in the dsRNA-treated group and 24.43 ± 1.57 in the CSBV-infected *A. cerana* groups (Figure 2). However, reference genes from the CSBV-infected group exhibited larger SD values, suggesting that studies should carefully consider when only using one gene as an internal reference for the quantification of genes related to CSBV in *A. cerana* (Figure 2D).

### Stability of the Candidate Reference Genes Under Different Viral Infections and dsRNA Treatment

**FIGURE 3:**
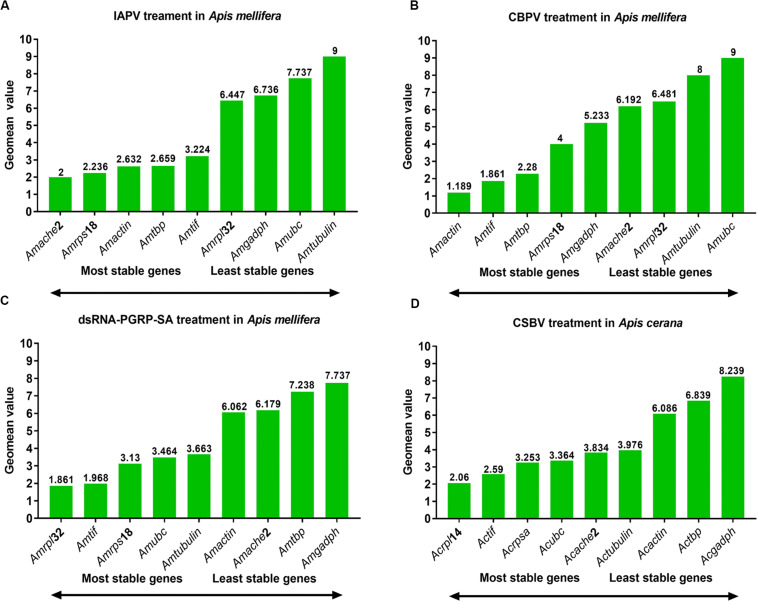
Expression stability of candidate reference genes analyzed by RefFinder. Expression stability of candidate reference genes during **(A)** IAPV infection, **(B)** CBPV infection, and **(C)** CSBV infection in *A. cerana* larvae as well as **(D)** honey bees treated with dsRNA-PGRP-SA.

The rankings for the expression stability of these 11 genes in honey bee under treatment with IAPV, CBPV, CSBV or dsRNA was analyzed using different algorithms (Table 1 and Figure 3). In the IAPV treatment group, *Amrps*18, *Amtif*, and *Amache*2 were considered the most stable genes with the lowest *M* value according to the geNorm and NormFinder analysis (Table 2). However, the ranking obtained with BestKeeper and Delta CT showed that *Amache*2, *Amtbp*, and *Am*β*-actin* were the top three stable candidate reference genes (Table 2). To identify the most suitable reference genes, the results of these four programs were integrated with RefFinder (He et al., 2019), which showed that the stability of these genes in *A. mellifera* treated with IAPV was as follows: *Amache*2, *Amrps*18, *Amactin*, *Amtbp*, *Amtif*, *Amrpl*32, *Amgadph*, *Amubc*, and *Am*α*-tubulin* (Figure 3A). However, *Amubc* and *Am*α*-tubulin* were ranked as the least stable genes in the IAPV-infected group.

**TABLE 2:** Gene expression stability ranked by the geNorm, NormFinder, and BestKeeper algorithms.

**Treatments**	**Gene**	**GeNorm**		**NormFinder**		**BestKeeper**			**delta-CT**	
		**M**	**R**	**SV**	**R**	**CV**	**SD**	**R**	**SD**	**R**
IAPV	*Am*β*-acti*n	0.749	4	0.02	3	2.14	0.432	3	0.588	3
	*Amrps*18	0.659	1	0.018	2	2.41	0.468	5	0.669	4
	*Amgadph*	0.820	5	0.077	6	2.77	0.729	6	0.996	7
	*Am*α*-tubulin*	1.373	8	0.267	8	3.22	0.999	9	1.332	9
	*Amubc*	0.839	6	0.101	7	2.66	0.737	7	0.985	6
	*Amrpl*32	0.871	7	0.038	4	4.13	0.850	8	1.041	8
	*Amtif*	0.697	2	0.014	1	1.73	0.437	4	0.726	5
	*Amtbp*	0.749	4	0.053	5	1.34	0.363	2	0.446	2
	*Amache*2	0.742	3	0.018	2	1.08	0.255	1	0.318	1
CBPV	*Am*β*-actin*	0.380	1	0.015	2	0.96	0.166	1	0.211	1
	*Amrps*18	0.430	3	0.015	2	1.64	0.285	4	0.669	8
	*Amgadph*	0.464	4	0.030	4	1.35	0.314	6	0.463	5
	*Am*α*-tubulin*	0.640	7	0.096	6	1.59	0.470	8	0.617	7
	*Amubc*	0.732	8	0.067	5	2.28	0.595	9	0.750	9
	*Amrpl*32	0.488	5	0.030	4	2.07	0.381	7	0.467	6
	*Amtif*	0.380	1	0.013	1	0.81	0.197	2	0.262	2
	*Amtbp*	0.407	2	0.030	4	0.85	0.218	3	0.263	3
	*Amache*2	0.507	6	0.021	3	1.32	0.287	5	0.376	4
dsRNA	*Am*β*-actin*	1.023	5	0.033	3	5.55	1.064	8	1.384	9
	*Amrps*18	1.152	7	0.049	4	5.55	1.064	8	0.963	4
	*Amgadph*	1.032	6	0.063	6	3.85	0.992	7	1.332	8
	*Am*α*-tubulin*	1.255	8	0.147	9	2.03	0.636	1	0.617	1
	*Amubc*	0.895	3	0.058	5	2.82	0.766	4	1.024	5
	*Amrpl*32	0.856	2	0.030	2	3.60	0.727	3	1.030	6
	*Amtif*	0.806	1	0.014	1	2.94	0.768	5	0.914	3
	*Amtbp*	1.032	6	0.080	7	3.45	0.946	6	1.263	7
	*Amache*2	0.925	4	0.082	8	2.97	0.707	2	0.843	2
CSBV	*Acacetin*	1.450	4	0.124	8	4.39	1.161	4	1.676	7
	*Acrpsa*	2.077	9	0.059	3	5.73	1.267	7	1.550	3
	*Acgadph*	1.580	6	0.108	7	6.73	1.514	9	1.852	9
	*Ac*α*-tubulin*	1.490	5	0.077	5	4.85	1.098	2	1.604	6
	*Acubc*	1.758	7	0.062	4	5.42	1.325	8	1.833	8
	*Acrpl*14	1.435	3	0.037	1	6.13	1.200	6	1.555	4
	*Actif*	1.371	1	0.053	2	4.86	1.187	5	1.574	5
	*Actbp*	1.769	8	0.178	9	4.22	1.149	3	1.526	2
	*Acache*2	1.428	2	0.099	6	3.82	1.093	1	1.467	1

*M, gene expression stability; R, ranking; SV, stability value; SD, standard deviation; CV, coefficient of variation.*

As shown in Table 2, *Amtif* and *Amtbp* were the top two stable candidate reference genes in *A. mellifera* infected with CBPV based on the ranking with geNorm, Delta-CT and BestKeeper. However, the ranking obtained with NormFinder was *Amtif*, *Amactin* and *Amrps*18, which was different from that obtained with geNorm. Based on the comprehensive analysis by RefFinder, the stability of these genes in *A. mellifera* treated with CBPV was as follows: *Am*β*-actin*, *Amtif*, *Amtbp*, *Amrps*18, *Amgadph*, *Amache*2, *Amrpl*32, *Amubc*, and *Am*α*-tubulin* (Figure 3B). Because *Amubc* and *Am*α*-tubulin* were ranked as the least stable genes in IAPV-infected and CBPV-infected *A. mellifera*, they were not recommended as reference genes for qRT-PCR analyses in honey bee infected with viruses (Figures 3A,B).

For *A. cerana* larvae infected with CSBV, *Actif*, *Acache*2, and *Acrpl*14 were the top three stable candidate reference genes based on the geNorm algorithms (Table 2). *Acrpl*14, *Actif*, and *Acrpsa* were the top three stable candidate reference genes according to NormFinder. However, the ranking obtained with BestKeeper showed that *Acache*2, *Ac*α*-tubulin* and *Actbp* were the top three stable candidate reference genes. Furthermore, the ranking obtained with the Delta CT method showed that *Acache2*, *Actbp*, and *Acrpsa* were the top three suitable genes (Table 2). The outcome from integration with RefFinder showed that the stability of the genes in *A. cerana* infected with CSBV was as follows: *Acrpl*14, *Actif*, *Acrpsa*, *Acubc*, *Acache*2, *Ac*α*-tubulin*, *Acacetin*, *Actbp*, and *Acgadph* (Figure 3C). However, according to their larger *M* values (1.3 to 2.0), none of the reference genes used in CSBV-infected *A. cerana* was stable (Table 2).

For *A. mellifera* treated with dsRNA-PGRP-SA and dsRNA-GFP, *Amtif*, *Amrpl*32 and *Amubc* were the top three stable candidate reference genes according to the geNorm algorithms (Table 2). *Amtif*, *Amrpl*32 and *Am*actin were the top three stable candidate reference genes based on NormFinder. However, the ranking obtained with BestKeeper showed that *Am*α*-tubulin*, *Amache*2, and *Amrpl*32 were the top three stable candidate reference genes. Moreover, the ranking by the Delta-CT method showed that *Am*α-tubulin, *Amache*2 and *Amtif* were the top three reference genes (Table 2). According to the RefFinder results, the stability of the genes in *A. mellifera* treated with dsRNA was as follows: *Amrpl*32, *Amtif*, *Amrps*18, *Amubc*, *Am*α-tubulin, *Amactin*, *Amache*2, *Amtbp*, and *Amgadph* (Figure 3D).

### Determination of the Optimal Number of Reference Genes

**FIGURE 4:**
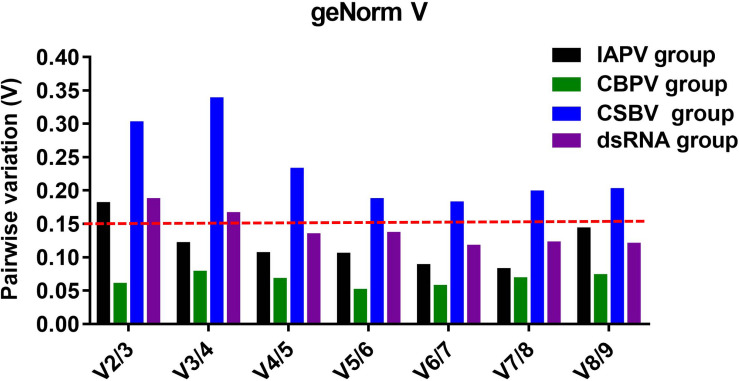
Determination of the optimal number of reference genes for normalization based on the pairwise variation values calculated by geNorm. The value of Vn/Vn + 1 < 0.15 means n should be the optimal number of reference genes selected for RT-qPCR analysis among IAPV-infected (black), CBPV-infected (green), and CSBV-infected (blue) honey bees, as well as honey bees exposed to dsRNA-PGRPSA (purple).

To determine the optimal number of reference genes, we used geNorm to calculate the pairwise variation. In the IAPV treatment group, V_3_/V_4_ < 0.15 indicated that three reference genes was sufficient to accurately normalize RT-qPCR data (Figure 4). Therefore, *Amache*2, *Amrps*18 and *Amactin* were considered to be a suitable combination of reference genes for RT-qPCR analyses in honey bee infected with IAPV. In the CBPV treatment group, V_2_/V_3_ < 0.15 indicated that two reference genes was sufficient to normalize RT-qPCR data. Thus, *Amactin* and *Amtif* were considered to be a suitable combination of reference genes for RT-qPCR. In contrast, in the CSBV treatment group, Vn/Vn + 1 > 0.15 indicated that more reference genes are required and should be combined for RT-qPCR data normalization. In the dsRNA treatment group, V_4_/V_5_ < 0.15 suggested that *rpl*32, *tif*, *rps*18, and *ubc* were a suitable combination of reference genes for RT-qPCR analyses in honey bee treated with dsRNA (Figure 4).

### Validation of the Selected Reference Genes

**FIGURE 5:**
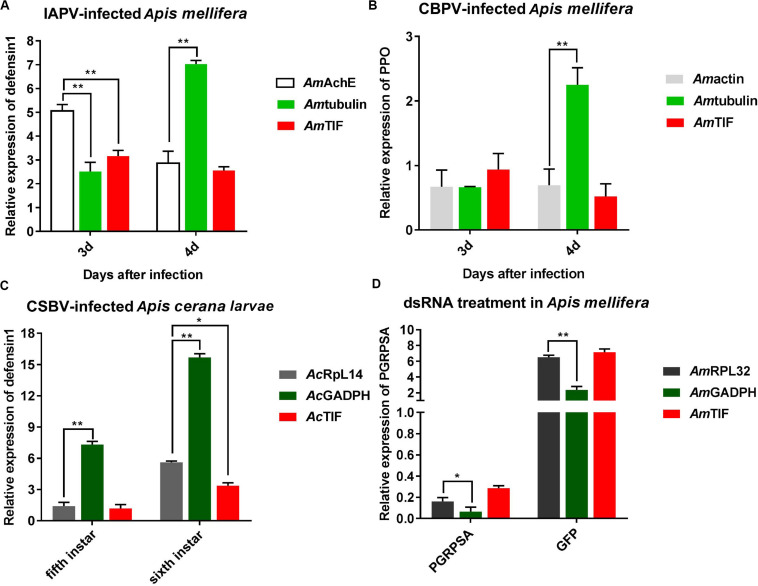
Validation of reference gene stability. **(A)** Relative expression levels of *defensin 1* in IAPV-infected honey bee. **(B)** Relative expression of *ppo* in CBPV-infected honey bee. **(C)** Relative expression of *defensin 1* in CSBV-infected honey bee larvae. **(D)** Relative expression of PGRP-SA in honey bees treated with dsRNA-PGRP-SA. Bars represent the means and standard deviations of three biological replicates. Asterisks indicate significant differences. (*P < 0.05 and **P < 0.01).

As shown in Figure 5A, the relative expression levels of the *defensin 1* and *ppo* genes varied based on the different reference genes. In the IAPV-infected group, the relative expression levels of *defensin 1* were downregulated from 5.1- to 2.9-fold and from 3.2- to 2.6-fold when *Amache*2 and *Amtif* were used as reference genes, respectively, while the expression levels of *defensin 1* were upregulated from 2.5- to 7-fold if *Am*α*-tubulin* was the reference gene. In the CBPV-infected group, the normalization of *ppo* expression levels to *Amactin* or *Amtif* as reference genes exhibited similar downregulated expression trends for both CBPV-infected and uninfected honey bees (Figure 5B). However, the relative expression levels of *ppo* were upregulated to 2.25-fold at 96 h after CBPV infection when *Am*α*-tubulin* was the reference gene (Figure 5B). In the CSBV-infected group, the relative expression levels of *defensin 1* were upregulated from 1.4- to 5.6-fold and from 1.2- to 3.4-fold in 5th and 6th CSBV-infected honey bee larvae when we used *Acrpl*14 and *Actif* as reference genes, respectively, while the relative expression levels of *defensin 1* were upregulated from 7.3- to 15.3-fold when *Acgadph* used as the reference gene (Figure 5C). In contrast, when *Acgadph* was used for normalization, the expression levels of *defensin 1* exhibited a significant difference from that of *Acrpl*14 (*P* < 0.01). In the dsRNA treatment group, the relative expression levels of *pgrpsa* were downregulated 0. 16-, 0. 06-, and 0.28-fold after 72 h of dsRNA-*pgrpsa* treatment when *Amrpl*32 (best), *Amgadph* (worst) and *Amtif* (modest) were used as the reference genes (Figure 5D), respectively, while the relative expression levels of *pgrpsa* were upregulated 6. 5-, 2. 4-, and 7.2-fold at 72 h after dsRNA-gfp treatment if we used *Amrpl*32, *Amgadph* and *Amtif* as the reference genes, respectively (Figure 5D). This result showed that normalization of *pgrpsa* expression using the best (*Amrpl*32) and modest (*Amtif*) reference genes exhibited nearly similar outcomes after dsRNA treatment, though its expression exhibited significant differences (*P* < 0.05 and *P* < 0.01) compared to those of *Amrpl*32 and *Amgadph* as reference genes (Figure 5D).

## Discussion

RT-qPCR is an important, simple and practical technique for assessing gene expression when compared to other quantitative methods such as Northern blotting, *in situ* hybridization and RNA-seq technology (Peng et al., 2018; Adeyinka et al., 2019; He et al., 2019). The application of accurate reference genes is crucial for the quantification of gene expression in *A. mellifera* and *A. cerana*. In our study, five algorithms were used to identify the stability of reference genes. *Ache*2, *rps*18, *actin*, *tbp*, and *tif* are suitable to normalize the gene expression levels in IAPV-infected *A. mellifera*. The combination of *actin* and *tif* is suitable for CBPV-infected *A. mellifera*, while the combination of *rpl*14, *tif*, *rpsa*, *ubc*, and *ache*2 as well as more reference genes is suitable for CSBV-infected *A. cerana* larvae. This result suggests that further studies should be performed to select candidate reference genes for CSBV infection. Moreover, *rpl*14, *tif*, *rps*18, *ubc*, and α*-tubulin* showed the most stable expression in *A. mellifera* under dsRNA treatment.

Our results showed that reference genes were different under different viral infection conditions. An increasing number of studies have reported that using a single reference gene to normalize RT-qPCR might be insufficient and inaccurate for the quantification of gene expression (Yang et al., 2018). As shown in our four experiments, the number of suitable reference genes was greater than two (Figure 4). Additionally, the stability of these reference genes was different under different experimental conditions. As determined by RefFinder (Figure 3), *tif* was a relatively stable reference gene under all of our experimental conditions. Actually, *tif* have been widely used as housekeeping genes for gene expression analysis during Bombyx mori Cytoplasmic Polyhedrosis Virus (BmCPV) and Bombyx mori Bidensovirus (BmBDV) infection (Guo et al., 2016). *Ache*2 was the most stable reference gene under IAPV infection, while β*-actin* was the most stable reference gene under CBPV infection. In contrast, α*-tubulin* exhibited the worst stability under IAPV and CBPV infection. *AcRpl*14 and *Amrpl*32 were the most stable reference genes under CSBV infection and dsRNA treatment, respectively, while *Acgadph* and *Amgadph* exhibited the worst stability under CSBV infection and dsRNA treatment, respectively. However, *gapdh* and *rps*18 were suggested to be the optimal reference genes for RT-qPCR-based under the labor-specific gene expression, bacterial infected and developmental gene expression in Western honey bee as shown in Table 2 (Scharlaken et al., 2008; Reim et al., 2013; Moon et al., 2018a). Previous studies have shown that some ribosome-associated genes have been used as stable internal reference genes for quantitative analysis (Wang et al., 2019). For instance, Freitas et al. (2019) found that *rpl*32, *rps*5, and *rps*18 were identified as suitable reference genes for the different tissues of stingless bees. Similarly, *rpl23* was identified the reliable reference gene in bumblebees challenged by IAPV (Niu et al., 2014; Table 3). Although our study showed that *rpl*32 displayed stable expression in *A. mellifera* under dsRNA treatment, it exhibited the worst stability during IAPV and CBPV infection in *A. mellifera* (Figure 3), while *rps*18 was the better reference gene in *A. mellifera* under dsRNA treatment or viral infection (Table 3).

**TABLE 3:** Comparison analysis on stability of candidate reference genes in stingless bees, bumblebee, *Apis mellifera* and *Apis cerana*.

**Bees**	**Condition**	**Most stable reference genes**	**References**
**Stingless bees** (*Frieseomelitta*	Development	*rpl32*, *rps18*	Freitas et al., 2019
varia, Melipona quadrifasciata,	Sex	*rpl32*, *rps18*, *rps5*, *tbp-af*	Freitas et al., 2019
and Scaptotrigona bipunctata)	Tissues, organs and body parts	*rps18*, *rps5*, *tbp-af*	Freitas et al., 2019
	Bacterial injection	*rps18*, *rpl32*, *tbp-af*	Freitas et al., 2019
	Pesticide exposure	*rps5*, *tbp-af*, *rpl32*, *rps18*	Freitas et al., 2019
***Bombus terrestris***	IAPV infection	*ppai*, *rpl23*, *ubi*	Niu et al., 2014
***Apis mellifera***	Abdomens	*rpl32*, *rps18*, *or gapdh*	Moon et al., 2018b
	Head	*rps18*, *gapdh*	Moon et al., 2018a
	Bacterial infection	*actin*, *rpS18*, *gapdh*	Scharlaken et al., 2008
	Development	*gapdh*, *rpl32*, *ef1*α*-f1*	Reim et al., 2013
	IAPV infection	*ache2*, *rps18*, *actin*, *tbp, tif*	This study
	CBPV infection	*actin*, *tif*, *tbp*, *rps18*	This study
	dsRNA treatment	*rpl32*, *tif*, *rps18*, *ubc*, *tubulin*	This study
***Apis cerana***	CSBV infection	*rpl14*, *tif*, *rpsa*, *ubc*, *ache2*, *tubulin*	This study

*Elongation factor 1 alpha-f1: *ef1*α*-f1*, peptidylprolyl isomerase A: ppia, polyubiquitin:UBI.*

Likewise, the stability of the same reference gene varied under different experimental conditions. Although actin is used as an internal control in most gene expression studies during honey bee viral infection, such as IAPV infection (Chen et al., 2014), CSBV infection (Shan et al., 2017), and CBPV infection (Coulon et al., 2017), *actin* exhibited poorer stability during CSBV infection (Figure 3). *Gadph*, the other most commonly used reference gene, also showed expression variability from the 2nd to 6th honey bee infected with CSBV infection as well as dsRNA treatment (Figure 3). Similarly, α*-tubulin*, another commonly used reference gene, exhibited worse stability under IAPV and CBPV infection. This might result due to interactions between α-tubulin and acetylation, as Zhang et al. (2016) found that acetylated α*-tubulin* will enhance viral titers by promoting fusion with viral inclusion bodies.

This study systematically analyzed 11 reference genes for RT-qPCR under viral infection in *A. mellifera* and *A. cerana*. These results will facilitate future studies in honey bee under different viral infection conditions.

## Data Availability Statement

The raw data supporting the conclusions of this manuscript will be made available by the authors, without undue reservation, to any qualified researcher.

## Author Contributions

CH and YD conceived the manuscript. YD, HZ, SY, LiZ, and LinZ performed the experiments. YD analyzed the data and wrote the manuscript. CH revised the manuscript. All authors contributed to the article and approved the submitted version.

## Conflict of Interest

The authors declare that the research was conducted in the absence of any commercial or financial relationships that could be construed as a potential conflict of interest.

## Supplementary Material

FIGURE S1 Sample collection and validation by RT-PCR. **(A)** IAPV was injected into newly emerged *A. mellifera* after 24, 48, 72, and 96 h. **(B)** CBPV was injected into newly emerged *A. mellifera* after 0, 24, 48, 72, and 96 h. **(C)** The larvae naturally infected with CSBV were validated from the 5th to 9th instar. **(D)** dsRNA-PGRP-SA and dsRNA-GFP were injected into newly emerged *A. mellifera* and validated at 24, 48, 72, and 96 h.

TABLE S1 Primer used for detection common viruses and PGRP-SA.

TABLE S2 Primer sequences and amplicon size for dsRNA synthesis of PGRP-SA and GFP.

TABLE S3 The standard curve for the quantification RT-qPCR of the reference genes.
