# Supplementary figures and images for: Screening and Validation of Reference Genes for RT-qPCR Under Different Honey Bee Viral Infections and dsRNA Treatment
Source: Front Microbiol. 2020 Jul 30;11:1715. doi: 10.3389/fmicb.2020.01715 (PMC7406718; doi:10.3389/fmicb.2020.01715)

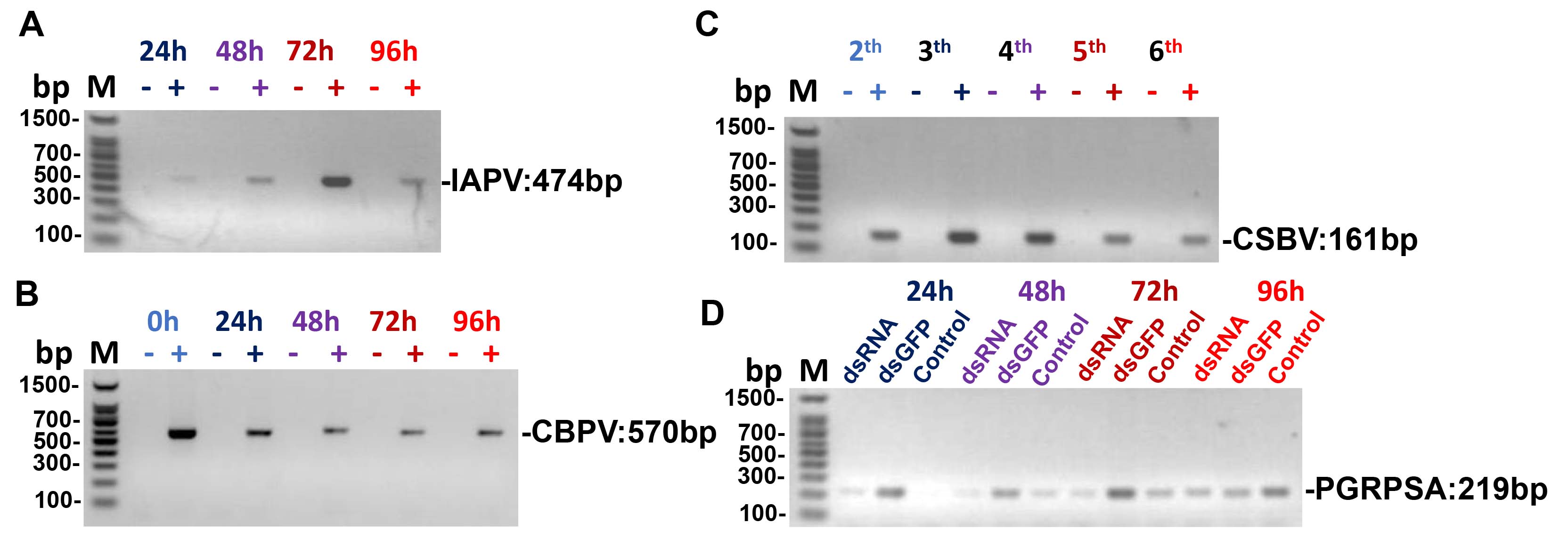

Supplement: FIGURE S1 — Sample collection and validation by RT-PCR. (A) IAPV was injected into newly emerged A. mellifera after 24, 48, 72, and 96 h. (B) CBPV was injected into newly emerged A. mellifera after 0, 24, 48, 72, and 96 h. (C) The larvae naturally infected with CSBV were validated from the 5th to 9th instar. (D) dsRNA-PGRP-SA and dsRNA-GFP were injected into newly emerged A. mellifera and validated at 24, 48, 72, and 96 h. [file Image_1.JPEG]
