# Supplementary material for: Screening and Validation of Reference Genes for RT-qPCR Under Different Honey Bee Viral Infections and dsRNA Treatment
Source: Front Microbiol. 2020 Jul 30;11:1715. doi: 10.3389/fmicb.2020.01715 (PMC7406718; doi:10.3389/fmicb.2020.01715)
Supplement: TABLE S1 — Primer used for detection common viruses and PGRP-SA. [file Table_1.DOCX]

**TABLE S1**∣Primer used for detection common viruses and PGRP-SA.

| **viruses** | **Viral name** | **Primer sequence (5’–3’)** | **Amplification length** |
| --- | --- | --- | --- |
| **IAPV** | **Israeli acute paralysis virus** | **F:AGACA CAATCACGGACCTCAC** | **474** |
|  |  | **R:AGATTT GTCTGTCT CCAGTG CAC** |  |
| **CBPV** | **Chronic bee paralysis virus** | **F:TCAGACAC GAATCT GATTAT TG** | **570** |
|  |  | **R:ACTACTAGAAACTCGTCGCTT CG** |  |
| **CSBV** | **Chinese sacbrood virus** | **F:CCTGGGAAGTTTGCTAGTATTTACG** | **161** |
|  |  | **R:CCTATCACATCCATCTGGGTCAG** |  |
| **PGRP-SA** | **peptidoglycan-recognition protein SA** | **F:TATATTGTCGGCGGAGATGAA** | **219** |
|  |  | **R:TCCGATATCATGCCAATTTAGGGT** |  |
| **BQCV** | **Black queen cell virus** | **F:TGGTCAGCT CCCACTACCTTA AAC** | **700** |
|  |  | **R:GCAACAAGAAGAAACGTA AA CAC** |  |
| **DWV** | **Deformed wing virus** | **F: GACTGAACCAAATCCGATGTC** | **376** |
|  |  | **R: TCTCAAGTTCGGGACGCATTC** |  |
| **ABPV** | **Acute bee paralysis virus** | **F:TTA TGT GTC CAG AGA CTG TAT** | **900** |
|  |  | **R:GCT CCT ATT GCT CGG TTT TTC** |  |
| **ALPV** | **Aphid lethal paralysis virus** | **F:GCGTACCATACTACTCACCATATTTAT** | **140** |
|  |  | **R:AGTTAATCCATAAAGTGCAATCTACAATAC** |  |
