# Supplementary material for: Screening and Validation of Reference Genes for RT-qPCR Under Different Honey Bee Viral Infections and dsRNA Treatment
Source: Front Microbiol. 2020 Jul 30;11:1715. doi: 10.3389/fmicb.2020.01715 (PMC7406718; doi:10.3389/fmicb.2020.01715)
Supplement: TABLE S2 — Primer sequences and amplicon size for dsRNA synthesis of PGRP-SA and GFP. [file Table_2.DOCX]

**TABLE S2**∣ Primer sequences and amplicon size for dsRNA synthesis of PGRP-SA and GFP.

| Gene name | Gene symbol | Primer sequence (5’–3’) | Amplicon  size (bp) | GenBank  accession no. |
| --- | --- | --- | --- | --- |
| *peptidoglycan-recognition protein SA* | *pgrpsa* | F:**TAATACGACTCACTATAGGGAGA**GGATACCCTAAATTGGCATGA | 254 | NM_001163715.1 |
|  |  | R:TAATACGACTCACTATAGGGAGATACCTCCGATTACCCGAACAT |  |  |
| *eGFP* | *gfp* | F:**TAATACGACTCACTATAGGGAGA**TTCATGGCCAACACT TGTCC | 268 | U17997; Clontech |
|  |  | R:**TAATACGACTCACTATAGGGAGA**CAAGAAGGACCATGTGGTC |  |  |

Bold letters denote promoter sequences of T7 RNA polymerase.
