# Supplementary material for: Screening and Validation of Reference Genes for RT-qPCR Under Different Honey Bee Viral Infections and dsRNA Treatment
Source: Front Microbiol. 2020 Jul 30;11:1715. doi: 10.3389/fmicb.2020.01715 (PMC7406718; doi:10.3389/fmicb.2020.01715)
Supplement: TABLE S3 — The standard curve for the quantification RT-qPCR of the reference genes. [file Table_3.DOCX]

**TABLE S3** ∣ The standard curve for the quantification RT-qPCR of the reference genes.

| Genes | The equation of standard curves | Amplification efficiency (E%) | Regression  Coefficient(R^2^) | Tm (℃) |
| --- | --- | --- | --- | --- |
| *Amrpl*32 | y=-3.2x+35.57 | 105.22 | 0.997 | 80 |
| *Amrps*18 | y=－3.49x+37.81 | 93.55 | 1 | 80 |
| *Amtbp* | y=－3.37x+38.63 | 98.05 | 0.999 | 80 |
| *Amtubulin* | y=－3.28x+36.46 | 101.62 | 0.998 | 78.5 |
| *Amgadph* | y=－3.38x+38.44 | 97.81 | 0.999 | 82.5 |
| *Amache*2 | y=-3.22x+37.39 | 104.75 | 0.999 | 84.5 |
| *Amtif* | y=-3.52x+41.85 | 92.51 | 0.996 | 77 |
| *Amubc* | y=-3.36x+37.55 | 98.32 | 0.999 | 81 |
| *Amactin* | y=-3.36x+35.37 | 98.40 | 0.998 | 81 |
| *Acrpl*14 | y=3.34x+36.16 | 99.28 | 0.999 | 82 |
| *Acrpsa* | y=－3.13x+32.89 | 108.55 | 0.997 | 80 |
| *Actbp* | y=-3.34x+35.88 | 99.29 | 1 | 79 |
| *Actubulin* | y=-3.26x+35.62 | 102.68 | 1 | 83 |
| *Acgadph* | y=-3.18x+33.97 | 106.38 | 0.999 | 82 |
| *Acache*2 | y=-3.58x+38.77 | 90.24 | 0.999 | 88.5 |
| *Actif* | y=-3.29x+36.64 | 101.32 | 0.999 | 80 |
| *Acubc* | y=-3.45x+36.33 | 95.04 | 0.999 | 82.5 |
| *Acactin* | y=-3.5x+37.04 | 93.23 | 0.998 | 88 |
